# Case Report: Spontaneous aneurysm of ductus arteriosus: A rare cause of hoarseness of voice in adults

**DOI:** 10.4103/0971-3026.43853

**Published:** 2008-11

**Authors:** Rajesh Gothi, Nitin P Ghonge

**Affiliations:** Body Imaging Division, Department of Radio-Diagnosis, Diwan Chand Imaging Research Center, 10 B, KG Marg, New Delhi-110 001, India

**Keywords:** Aneurysm of ductus arteriosus, hoarseness of voice, left recurrent laryngeal nerve palsy in adults

## Abstract

Ortner's syndrome (left recurrent laryngeal nerve palsy caused by cardiovascular pathology) is described in literature as occurring secondary to a variety of conditions. Spontaneous aneurysm of ductus arteriosus is a rare cause of this condition. We present a case where an adult patient with an aneurysm of the ductus arteriosus presented for the first time at the age of 62 years with hoarseness of voice secondary to left recurrent laryngeal nerve palsy.

## Case Report

A 62-year-old man presented with hoarseness ofvoice of 3 months' duration. The clinical history was negative for any major illness in the past. There was no history of any cardiovascular ailment during childhood. Indirect laryngoscopy showed left vocal cord palsy, without demonstrating any definite laryngeal mass lesion. 

**Figure 1 (A, B):**
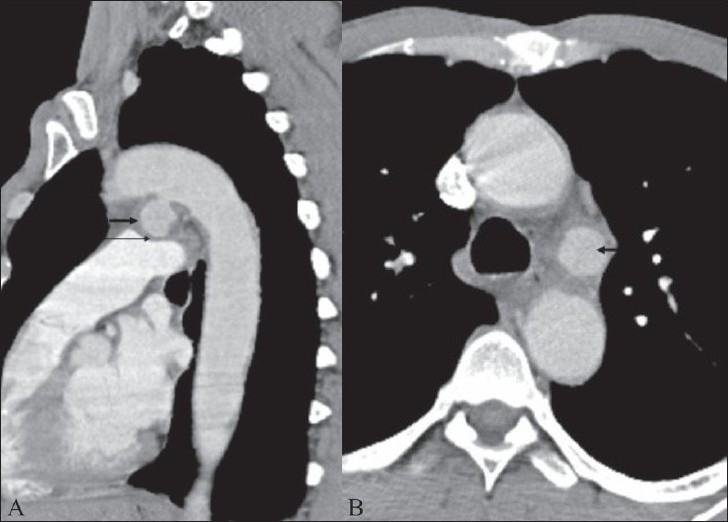
Sagittal reformatted (A) and axial (B) contrastenhanced CT scan of the chest shows a wide-necked luminal outpouching along the inferior aspect of the aortic arch (thick arrow) in the aorto-pulmonary window. The main pulmonary artery shows mild dilatation and indentation by the aneurysm in its distal part (thin arrow). The thoracic aorta shows normal caliber without any significant atherosclerotic changes. The ‘three-vessel’ appearance seen in B is also referred to as the ‘triple-star sign.’

A CT scan of the chest showed a luminal out-pouching along the inferior surface of the aortic arch. The characteristic location of the out-pouching in the aortopulmonary window suggested the diagnosis of a ductus arteriosus aneurysm. The saccular aneurysm measured approximately 3 cm in diameter and showed a wide neck, without any intraluminal thrombus. The aneurysm showed a distinct bulge toward the main pulmonary artery and appeared thick-walled [Figure 1a]. There were no signs of contrast extravasation. There was mild indentation over the adjacent main pulmonary artery without any definite mass effect on the adjacent airways. Mild dilatation of the main pulmonary artery was however noted [Figure 1b]. No definite pleuroparenchymal lesion in the lung or any cardiac lesion was identified. No significant atherosclerotic changes were identified in the thoracic aorta.

The sections through the neck did not reveal any laryngeal pathology. In view of the patient's symptoms, surgery was advised. The patient refused surgery and is presently on regular follow-up.

## Discussion

Ductus arteriosus is a normal anatomic structure that provides communication between the systemic and pulmonary circulations during fetal life and closes soon after birth. An indentation of the aortic wall at the site of insertion of the obliterated ductus arteriosus is seen in approximately 9–26% of adults on angiography studies and is referred to as a ductus diverticulum or bump.[1] 

Aneurysm of the ductus arteriosus may occur either spontaneously or may follow surgical treatment of a patent ductus arteriosus.[2] Spontaneous aneurysm of the ductus arteriosus (SADA) is an uncommon occurrence, with only 34 reported cases in the Japanese literature till 2002.[3] The Japanese literature accounts for a major proportion of the reported cases in adults.[4] 

SADA presenting in adults usually shows an obliterated pulmonary end of the ductus, unlike aneurysms in the pediatric age-group which occur in an open ductus arteriosus. The presence of concomitant hypertension can be a contributory factor. Connective tissue disorders such as Marfan syndrome and Ehlers-Danlos syndrome are known to predispose to ductus arteriosus aneurysms as well.[5,6] 

Hoarseness of voice, cough, anorexia, and chest pain are common presenting symptoms in adults and may be secondary to involvement of the adjacent organs and nerves. Hoarseness of voice occurs due to compression of the recurrent laryngeal nerve as it courses through the aorto-pulmonary window.

Ortner's syndrome was first described by Ortner[7] in 1897, when he reported the occurrence of paralysis of the left recurrent laryngeal nerve in a patient with mitral stenosis. However, based on anatomic studies, Fetterolf and Norris[8] were the first to show that the recurrent laryngeal nerve must be compressed in the aortopulmonary window between the left pulmonary artery, the aortic arch, and the ligamentum arteriosum to produce clinical symptoms of left recurrent laryngeal nerve palsy. In our case, the hoarseness of voice had a delayed onset, despite the long-standing presence of the ductus aneurysm. It is possible that concomitant dilatation of the main pulmonary artery may have contributed to left recurrent laryngeal nerve compression. 

Radiologically, SADA can present as a mass lesion in the aortopulmonary window and can be visualized on chest radiographs, CT scan, and MRI. Contrast-enhanced CT scan is the optimal imaging modality to establish the diagnosis.[9] The axial CT image through the aortopulmonary window shows the ductus aneurysm as a third vessel displaying arterial-phase contrast, apart from the ascending and the descending aorta. This is often referred to as the ‘triple-star sign.’[10] According to the criteria suggested by Cruickshank,[11] ductus arteriosus aneurysm should be diagnosed only when (a) the aorta does not reveal any significant arteriosclerotic process in the area, (b) the aneurysm shows a definite bulge towards the ductus and/or the pulmonary artery, and (c) the ductus arteriosus shows occlusion in adults, being represented by a closed fibrous strand. Our case appears to satisfy these criteria. 

SADA should also not be mistaken for a ductus diverticulum (‘ductus bump’). The latter is a small conical bulge along the posteroinferior aspect of the aortic arch and is usually incidental and does not cause any compression of the recurrent laryngeal nerve. The diagnosis of aneurysm of ductus arteriosus should therefore be made with caution.[12] 

Rupture of the aneurysm is reported to be the commonest complication in adults.[2] Erosion into adjacent mediastinal structures (pericardium, bronchi, and esophagus), endocarditis, and thrombosis have also been reported.[2,4] 

Thus, SADA is an uncommon but important cause of left recurrent laryngeal nerve palsy in adults. 
